# Frontal electroencephalogram based drug, sex, and age independent sedation level prediction using non-linear machine learning algorithms

**DOI:** 10.1007/s10877-020-00627-3

**Published:** 2020-12-14

**Authors:** S. M. Ramaswamy, M. H. Kuizenga, M. A. S. Weerink, H. E. M. Vereecke, M. M. R. F. Struys, S. Belur Nagaraj

**Affiliations:** 1 Department of Anaesthesiology, University of Groningen, University Medical Center Groningen, Groningen, The Netherlands; 2 Department of Clinical Pharmacy & Pharmacology, University of Groningen, University Medical Center Groningen, Groningen, The Netherlands; 3 Department of Anaesthesiology and Reanimation, AZ St.-Jan Brugge Oostende AV, Brugge, Belgium; 4 Department of Basic and Applied Medical Sciences, Ghent University, Ghent, Belgium

**Keywords:** Anaesthesia, Electroencephalogram, Medical informatics, Consciousness Monitors, Machine learning

## Abstract

Brain monitors which track quantitative electroencephalogram (EEG) signatures to monitor sedation levels are drug and patient specific. There is a need for robust sedation level monitoring systems to accurately track sedation levels across all drug classes, sex and age groups. Forty-four quantitative features estimated from a pooled dataset of 204 EEG recordings from 66 healthy adult volunteers who received either propofol, dexmedetomidine, or sevoflurane (all with and without remifentanil) were used in a machine learning based automated system to estimate the depth of sedation. Model training and evaluation were performed using leave-one-out cross validation methodology. We trained four machine learning models to predict sedation levels and evaluated the influence of remifentanil, age, and sex on the prediction performance. The area under the receiver-operator characteristic curve (AUC) was used to assess the performance of the prediction model. The ensemble tree with bagging outperformed other machine learning models and predicted sedation levels with an AUC = 0.88 (0.81–0.90). There were significant differences in the prediction probability of the automated systems when trained and tested across different age groups and sex. The performance of the EEG based sedation level prediction system is drug, sex, and age specific. Nonlinear machine-learning models using quantitative EEG features can accurately predict sedation levels. The results obtained in this study may provide a useful reference for developing next generation EEG based sedation level prediction systems using advanced machine learning algorithms.

**Clinical trial registration:** NCT 02043938 and NCT 03143972.

## Introduction

Optimal sedation level management is critical for a healthy outcome of patients undergoing surgical procedures/ in intensive care units which otherwise can lead to unwanted neurological and cardiovascular complications [1–4]. In recent decades, developing electroencephalogram (EEG) based sedation level monitoring techniques has been an active area of research and many such techniques have already been developed [5–8]. However, their performance is limited due to drug specificity and inter- (and intra-) subject variability [5, 7, 9, 10]. Neurophysiological distinctions [11], age [12] and sex-dependent EEG changes [13] between sedation drugs highlight the need for more robust techniques to monitor sedation levels.

To overcome the limitation of drug specificity, in our preliminary work [14], we developed a machine learning framework to design a drug-independent sedation level monitoring system using quantitative features derived from the frontal EEG. We developed this framework using a traditional logistic regression model which showed promising results in estimating sedation levels using pooled data from healthy volunteers during propofol, sevoflurane, and dexmedetomidine infusion. In the current study, we performed the following improvements to our previous work: (i) compared the performance of several nonlinear machine learning algorithms to predict sedation levels on a large EEG dataset of 204 EEG recordings, (ii) included remifentanil as an additional drug to the analysis and evaluated the stability of machine learning algorithms, and (iii) evaluated the robustness of the proposed framework across different age groups and sex. Our primary goal in this study was to develop a robust and reliable real-time automatic sedation level prediction system that is invariant across all conditions.

## Methods

### Ethics statement

The current study received ethical approval from the “The Independent Ethics Committee” (Medisch Ethische Toetsings Commissie) of the Foundation ‘Evaluation of Ethics in Biomedical Research’ (Stichting BEBO), Assen, The Netherlands.

### Dataset

A detailed description of the experimental protocol and EEG recordings have been described in full in the original studies [15, 16]. However, the main methodological topics of both studies with a direct relevance for this reanalysis, are recapitulated here. Information on the trial design and sample size calculation can be found in the previous studies [15, 16]. In general, we used an adaptive trial design and selected specific drug conentrations and number of volunteers in order to obtain an accurate level of information on the various dose-response relationships and/or drug interaction surfaces.

#### Propofol, sevoflurane and remifentanil EEG recordings

Thirty six age and gender stratified healthy volunteers (American Society of Anesthesiologists Class I) were included in this study (Table 1 of the online supplements [15]). Each age group (respectively 18–35, 36–55 and 56–70 years of age) contained 12 participants of which 6 females and 6 males each. During sessions that included the administration of remifentanil, the participants were also stratified to either a target effect-site concentration of remifentanil (Ce_REMI_) of 2 versus 4 ng/ml that was maintained throughout the study duration. Exclusion criteria were weight less than 70% or more than 130% of ideal body weight, pregnancy, diseases involving the neurological, cardiovascular, pulmonary, gastric, and endocrinological system, and recent use of psycho-active medication or intake of more than 20 g of alcohol daily.

**Table 1:** Summary of AUC’s (mean AUC (95% CI)) obtained for each model with (propofol, sevoflurane, dexmedetomidine and remifentanil) and without the inclusion of remifentanil (propofol, sevoflurane, dexmedetomidine). The performance of ensemble tree with bagging outperformed other machine learning models and was stable after the inclusion of remifentanil

Model	AUC		*P* value
	Without remifentanil	With remifentanil	
EN-LR	0.89 (0.81–0.92)	0.85 (0.80–0.88)	0.02
SVM-G	0.85 (0.77–0.88)	0.84 (0.75–0.89)	0.04
RF	0.83 (0.76–0.87)	0.82 (0.75–0.88)	0.06
ET-B	0.88 (0.85–0.91)	0.87 (0.84–0.89)	0.15

Abbreviations: *EN-LR* = elastic net logistic regression; *SVM-G* = support vector machine with Gaussian kernel; *RF* = random forest; *ET-B* = Ensemble tree with bagging

Each volunteer participated in four sessions of anesthesia with different drug combinations in a random order, with a minimal interval of 1 week in between sessions. The drug combinations administered were: “propofol alone”, “sevoflurane alone”, “propofol combined with remifentanil”, and “sevoflurane combined with remifentanil”. Propofol and remifentanil were administered through a Fresenius Base Primea docking station carrying two Fresenius Module DPS pumps (Fresenius-Kabi, Bad Homburg, Germany) that were controlled by a computer-controlled drug delivery and data collection software package (RUGLOOPII software (Demed, Temse, Belgium)). The effect-site concentration of propofol (Ce_PROP_) and remifentanil (Ce_REMI_) are calculated using the pharmacokinetic-dynamic (PKPD) model of respectively Schnider et al. [17] and Minto et al. [18]. The end-tidal vapor pressure of sevoflurane (ET_SEVO_) was titrated using the proprietary closed loop algorithm of the Zeus® ventilator (Software version 4.03.35, Dräger Medical, Lübeck, Germany).

The oxygen saturation (measured by pulse oximetry), electrocardiogram (ECG) and intermittently measured non-invasive blood pressure at 1-min intervals were monitored using a Philips IntelliVue MP50 monitor (Philips Medizin Systeme, Boeblingen, Germany). End-tidal sevoflurane (ET_SEVO_), carbon dioxide and oxygen concentration were monitored using a gas-analyzer of the anesthesia ventilator.

Raw EEG was collected from a standard 10–20 electrode montage, using a 16 channel Neuroscan® EEG monitor (Compumedics USA, Limited, Charlotte, NC, USA) and stored on a laptop computer running SCAN4 proprietary recording software (Compumedics, Charlotte, USA) at a sampling frequency of 5Khz.

In each session, the volunteers kept breathing spontaneously through a tight-fitting face mask connected to the anesthesia ventilator (Zeus®, Software version 4.03.35, Dräger Medical, Lübeck, Germany) although some additional respiratory support was required at deeper levels of hypnotic drug effect. After 2 min of baseline monitoring, a “staircase” step-up and step-down infusion of anesthetic drugs was administered. Ce_PROP_ was titrated in consecutive steps towards 0.5, 1, 1.5, 2.5, 3.5, 4.5, 6 and 7.5 μg/mL. For sevoflurane the ET_SEVO_ targets were 0.2, 0.5, 1.0, 1.5, 2.5, 3.5, 4, 4.5 vol%. The upwards steps were continued till a significant burst suppression ratio (>40%) was observed on the electroencephalogram. After that, a downward staircase was initiated using identical targets in reverse order. For sessions with remifentanil, Ce_REMI_ was targeted 2 min before administration of propofol or sevoflurane, at the randomized target of 2 or 4 ng/ml, and maintained throughout the study. After each change in effect-site target, a 12 min equilibration time was maintained before assessing the clinical sedation level using the Modified Observer’s Assessment of Alertness/Sedation (MOAA/S) scale. [19]

#### Dexmedetomidine, remifentanil EEG recordings

In this study [16], thirty volunteers were included and stratified according to age- and sex into 3 categories, respectively 18–34, 35–49 and 50–70 years. Written informed consent was obtained from each volunteer before recording EEG with similar exclusion criteria mentioned in the previous section. Each volunteer underwent two study sessions with at least 1 week in between.

Vital signs were monitored using the IntelliVue MP70 Patient Monitor, (Philips, Amsterdam, the Netherlands). A 20-gauge arterial cannula was placed for blood sampling and hemodynamic monitoring (EV1000 Monitor with FloTrac sensor, Edwards Lifesciences, Irvine, California, USA). Volunteers were connected to the ventilator (Zeus Infinity C500 ventilator, Dräger Medical, Lübeck, Germany) using a tight-fitting face mask. The cerebral drug effect was measured using 17-channel electroencephalography (EEG), with a BrainAmp DC32 amplifier and a Brainvision recorder (Brain Products GmbH, Gilching, Germany) recorded at a sampling rate of 5 kHz. In addition, we used a Sedline® PSI sensor (Masimo corporation, Irvine, CA, USA) with six electrodes that was specifically modified by the manufacturer to allow simultaneous measurements of patient state index while capturing raw signals in high resolution (5 kHz) using the Neuroscan EEG monitor.

On the first study day, volunteers received stepwise increasing effect-site concentrations of dexmedetomidine (Ce_DEX_) of respectively 1, 2, 3, 5 and 8 ng/ml as calculated by the PKPD model of Hannivoort and Colin et al., using the effect site prediction based on the MOAA/S observations. [20] For the first 3 infusion targets, the infusion rate was limited to 6 μg/kg/h and for the highest two targets to 10 μg/kg/h in order to avoid hypertensive reactions as seen with bolus administration of dexmedetomidine. On the second study day, subjects first received a stepwise increasing infusion of Ce_REMI_ targets, as calculated by the PKPD model of Eleveld et al. [21], of respectively 1, 2, 3, 5 and 7 ng/ml. After washout of remifentanil, a Ce_DEX_ of 2 ng/ml was administered and maintained while increasing targets of Ce_REMI_, set respectively to 0.5, 1.0, 1.5, 2.0, 2.5, 3.0 and 4.0 ng/ml. Drug infusion was stopped after completion of all infusion steps or when one of the safety criteria was met. Safety criteria were: (1) a change of more than 30% in mean arterial blood pressure compared to baseline for more than 5 min, (2) a heart rate < 40 bpm lasting more than 5 min, (3) a change in cardiac rhythm or conduction, (4) any other safety reason (decided by the attending anesthesiologists/researchers). All observations of responsiveness were done by three anesthesiologists-researchers: HEMV, MASW and Koen Reyntjens [15]. During the recovery phase, all drug administration was stopped and measurements and monitoring continued until the volunteer was fully recovered from anesthesia and met discharge criteria of the post anesthesia care unit.

The MOAA/S score was tested at baseline, before each increase of Ce_REMI_ target (after maintaining an appropriate equilibration time) and every 2 min during the first 30 min of recovery, and every 10 min thereafter. In both studies prior to the measurements, the electrode impedance was tested and optimized if needed (e.g. by adding extra lubrification gel on a high impedance electrode). After the last measurement we retested the electrode impedance to confirm a maintained adequacy of impedance during the measurements. However, during the study phase, the intermittent automatic impedance checks were switched off to avoid signal irregularities.

In total, 204 EEG recordings from 66 healthy volunteers were used for analysis in this study. We used EEG recordings from Neuroscan recorder for propofol and sevoflurane; Brainvision recorder for dexmedetomidine in this study. Only the four frontal EEG channels, re-referenced in bipolar montage: Fp1 – F7 and Fp2 – F8, were used for developing the prediction model. We bandpass filtered the raw signal (using a zero-phase second order Butterworth bandpass filter) between 0.5 – 25 Hz and resampled to 250 Hz. For this study, we performed a binary classification between two MOAA/S subgroups: awake [MOAA/S 5 and 4] versus sedated [MOAA/S 1 and 0], discarding the remaining MOAA/S scores.

### Development of the sedation level prediction system

**Fig. 1:**
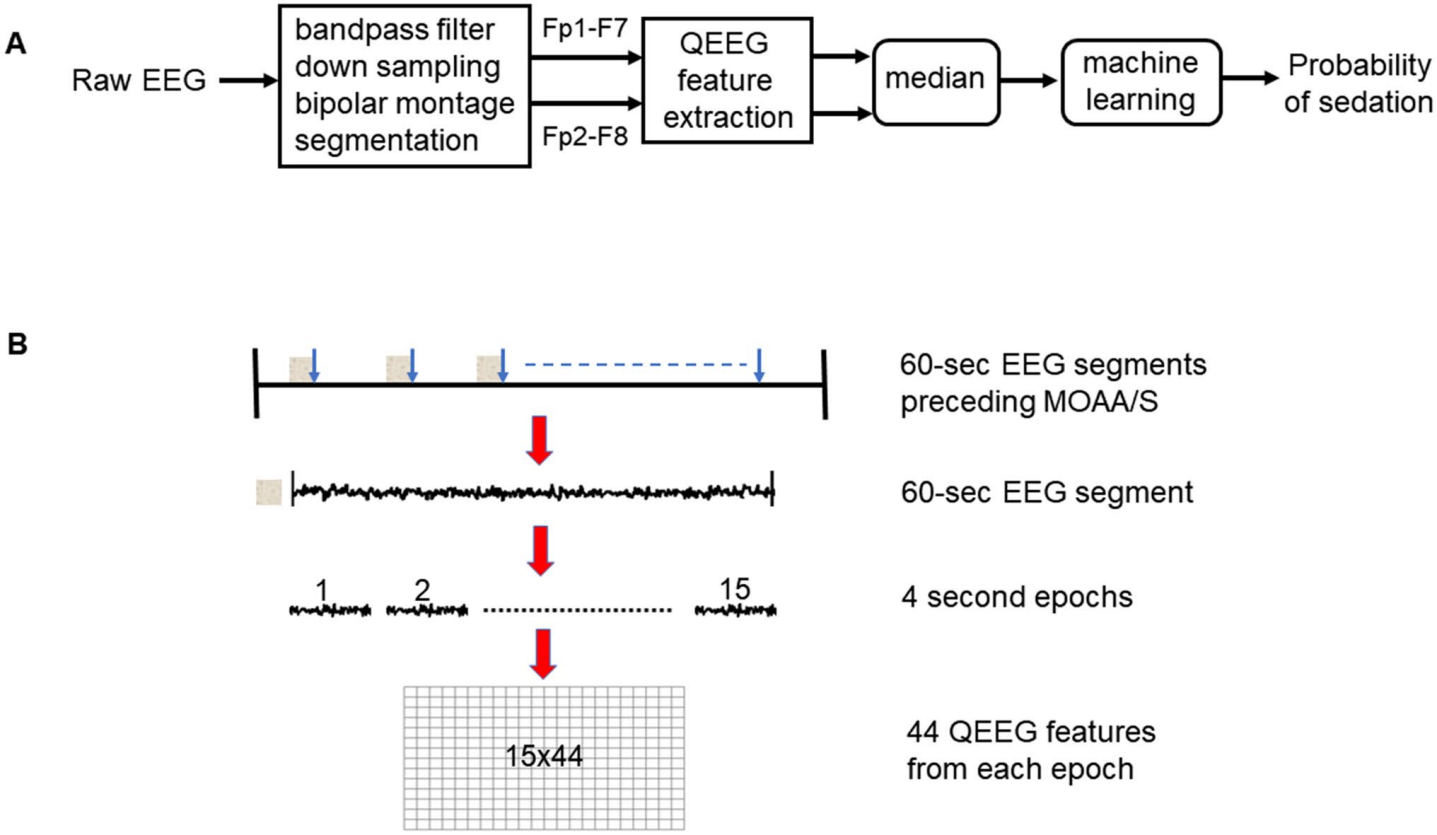
(**a**) Architecture of the proposed sedation level estimator, and (**b**) Illustration of the EEG epoch selection, segmentation and feature extraction process. One-minute EEG segments preceding the time of MOAA/S assessments were used for the analysis. Each segment was further divided into non-overlapping 4 s short EEG epochs and 44 QEEG features were extracted from each 4 s epoch

Fig. 1a shows the architecture of the proposed sedation level prediction system. From the downsampled signals, we extracted one minute EEG segments preceeding the MOAA/S assessments with an assumption that they correspond to the assessed MOAA/S score. Each one minute EEG segment was further divided into 4 s small duration epochs for further analysis (see Fig. 1b). EEG epochs with absolute amplitude >500 μV (corresponding to movement artifacts) and 0 μV (corresponding to flat EEG artifacts) were excluded for further analysis. Similar to our previous work [14], we extracted following 44 quantitative EEG (QEEG) features from each 4 s EEG epoch in this study:

- *Time domain* – (1) Nonlinear energy operator, (2) Activity (1st Hjorth parameter), (3) Mobility (2nd Hjorth parameter), (4) Complexity (3rd Hjorth parameter) [22], (5) Root mean square (RMS) amplitude, (6) Kurtosis, (7) Skewness, (8–11) mean, standard deviation, skewness and kurtosis of amplitude modulation (AM) [23], (12) Burst suppression ratio/min (BSR) [24];
- *Frequency domain* – (13) *P*_*δ*_=mean power in delta band (0.5–4 Hz), (14) *P*_*θ*_=mean power in theta band (4–8 Hz), (15) *P*_*α*_=mean power in alpha band (8–12 Hz), (16) *P*_*σ*_=mean power in spindle band (12–16 Hz), (17) *P*_*β*_=power in beta band (16–25 Hz), (18) *P*_*T*_=total spectral power (0.5–25 Hz), (19–23) *P*_*δ*_/*P*_*T*_, *P*_*θ*_/*P*_*T*_, *P*_*α*_/*P*_*T*_, *P*_*σ*_/*P*_*T*_, *P*_*β*_/*P*_*T*_, (24–27) *P*_*δ*_/*P*_*θ*,_*P*_*α*_/*P*_*θ*_, *P*_*σ*_/*P*_*θ*_, *P*_*β*_/*P*_*θ*_, (28–30) *P*_*α*_/*P*_*θ*_, *P*_*σ*_/*P*_*θ*_, *P*_*β*_/*P*_*θ*_, (31–34) mean, standard deviation, skewness and kurtosis of frequency modulation (FM) [23] (35) spectral edge frequency, (36) peak frequency;
- *Entropy domain* – (37) Singular value decomposition entropy [25], (38) spectral entropy [26], (39) state entropy [27], (40) sample entropy [27], (41) Renyi entropy [28], (42) Shannon entropy [29], (43) permutation entropy [30], (44) fractal dimension [31].

We extracted these features separately for each bipolar frontal montage channel and then obtained a median across channels to combine the channel information. These features were then used to train the machine learning algorithm to obtain the probability of the sedated state for each 4 s EEG epoch.

### Metrics

We used the area under the receiver operator characteristic curve (AUC) to evaluate the model performance. In addition, we also report sensitivity, specificity, F1-score for the best performing machine learning model.

### Machine learning model development

**Fig. 2:**
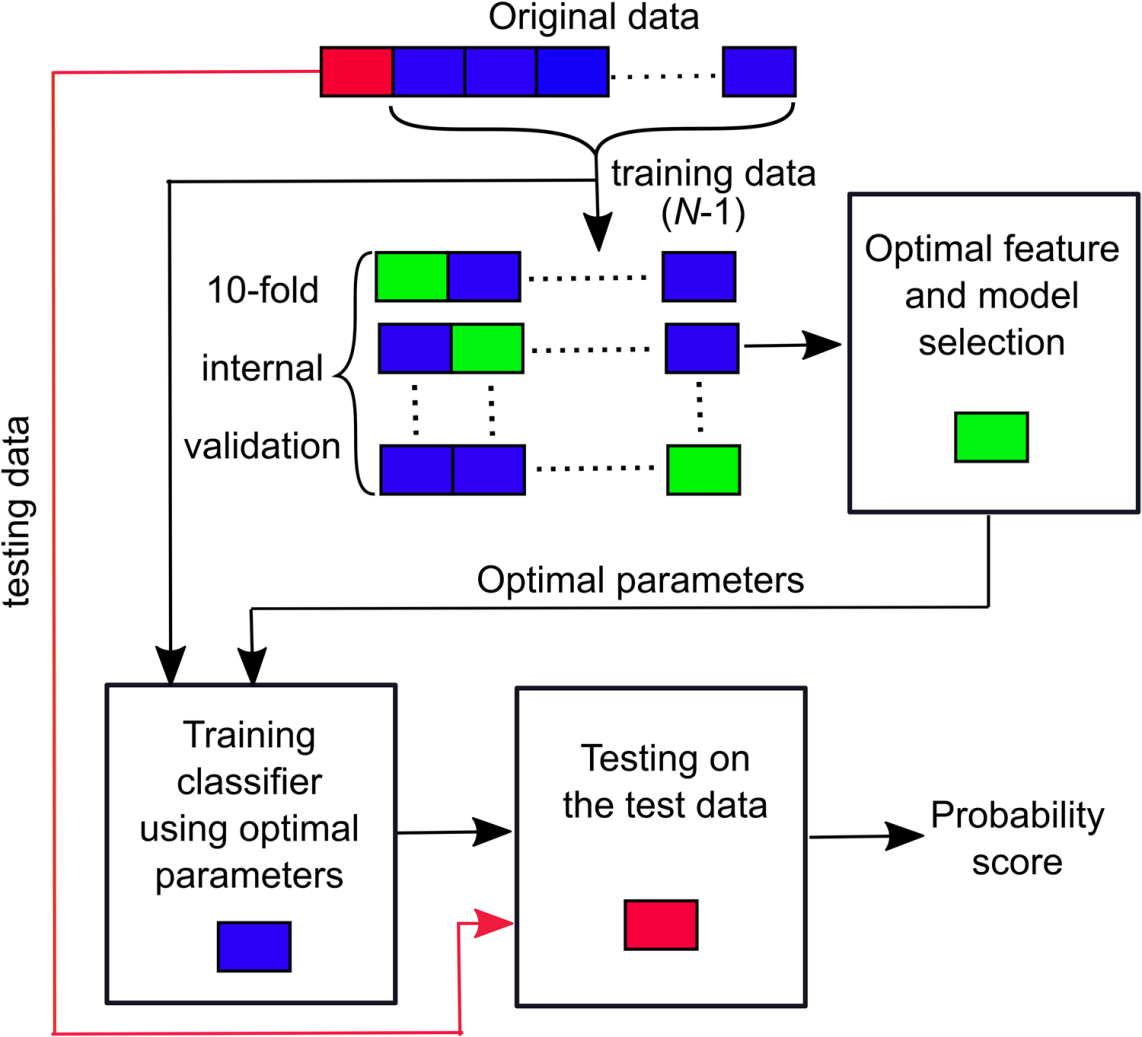
Illustration of the cross-validation strategy used in this study. A 10-fold cross validation using training data was used for model hyperparameters and feature selection and leave-one-subject-out cross validation was used to predict the sedation level for each subject

In this study, we evaluated the performance of four machine learning algorithms: elastic net logistic regression (EN-LR) [32], support vector machine with Gaussian kernel (SVM-G) [33], random forest (RF) [34], and Ensemble tree with bagging (ET-B) [35] that are commonly used for binary classification problems. We evaluated the performance of the proposed system using a leave-one-out cross-validation technique i.e. we divided the data into *N-*1 folds. In each iteration, we used *N*-1 EEG recordings for training the machine learning model and the left-out unseen recording for testing, resulting in a total of *N* iterations. In each fold, features in the training data were Z-score standardized (by subtracting the mean and dividing by the standard deviation) and the testing data features were normalized with respect to the Z-score normalization factor of the training data before using them for classification. We performed grid search to identify the optimal hyper-parameters of these models (summarized in Table 3) through 10-fold cross-validation within the training data and the final optimal model was then used to estimate the sedation level probability on the testing data. This was repeated until each data was used once for testing and is illustrated in Fig. 2.

First, we performed binary classification to differentiate between awake and sedated state using pooled dataset during propofol, sevoflurane and dexmedetomidine infusion. Then we added remifentanil data to this pooled dataset to evaluate the robustness and stability of the machine learning models. By this way we identified the machine learning model that is invariant after the addition of new drug (remifentanil in this case).

For significance analysis, we used Analysis of Variance (ANOVA) with the Tukey Honest Significant difference test. All tests were two-sided with alpha = 0.05. All of the coding and analysis was performed using the MATLAB 2018a scripting language (Natick, USA). All experiments were performed on a local computer with windows 10 platform, Intel Xeon 4116 processor and 32GB RAM. The overall time spent to extract these features from a 4 s epoch was 0.5 s and prediction of a sedation probability using a trained model was 0.05 s.

## Results

All results are reported as mean (95% confidence interval) unless stated otherwise. 95% confidence interval was obtained using bootstrapping with 1000 bootstraps.

### Performance of individual QEEG features

**Fig. 3:**
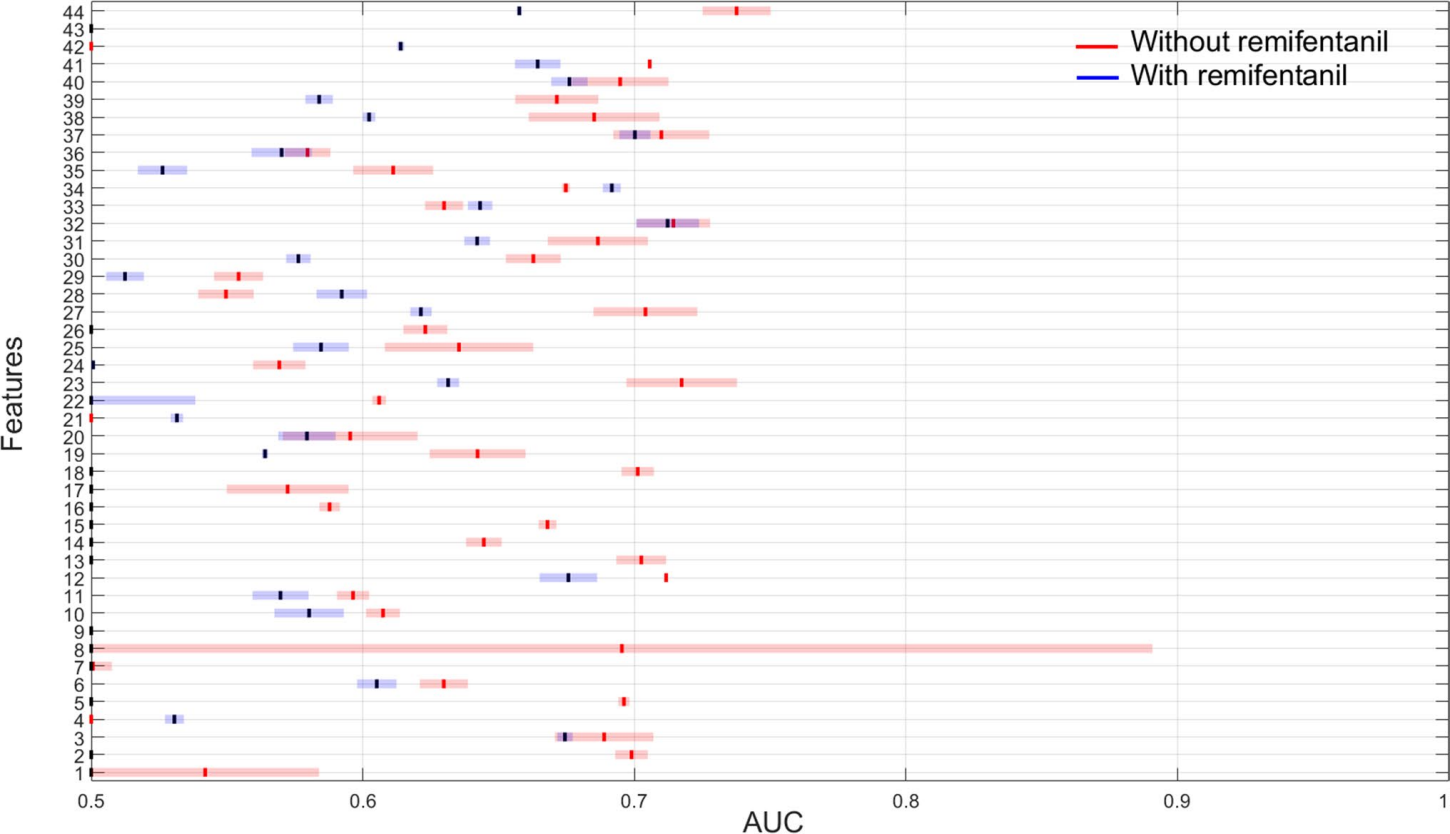
The distribution of AUC’s for individual features across all drugs to discriminate between awake and sedated EEG epochs with (propofol, sevoflurane, dexmedetomidine and remifentanil) and without remifentanil (propofol, sevoflurane, dexmedetomidine). The performance of all features significantly dropped after the addition of remifentanil. Here the vertical solid line indicates mean AUC and horizontal bar refers to standard deviation. X-axis corresponds to features: 1–12 = time domain, 13–36 = frequency domain and 37–44 = entropy domain features

Figure 3 shows the performance of individual features to discriminate between awake and sedated states with (propofol, sevoflurane, dexmedetomidine and remifentanil) and without remifentanil (propofol, sevoflurane, dexmedetomidine). Interaction with remifentanil significantly dropped the performance of all features. Fractal dimension provided the highest AUC of 0.74 (0.71–0.75) without remifentanil and dropped to 0.66 (0.64–0.68) after the addition of remifentanil.

### Performance of machine learning models

The performance of different machine learning models to predict sedation levels using the proposed architecture is summarized in Table 1. All models had AUC’s above 0.8 without remifentanil but the AUC’s dropped significantly when interacted with remifentanil. However, the performances of the tree based methods were not sensitive to the addition of remifentanil and the ET-B model achieved the highest AUC of 0.88 (0.84–0.89). All subsequent results will be based on the performance of ET-B including remifentanil.

### Discriminative features

**Fig. 4:**
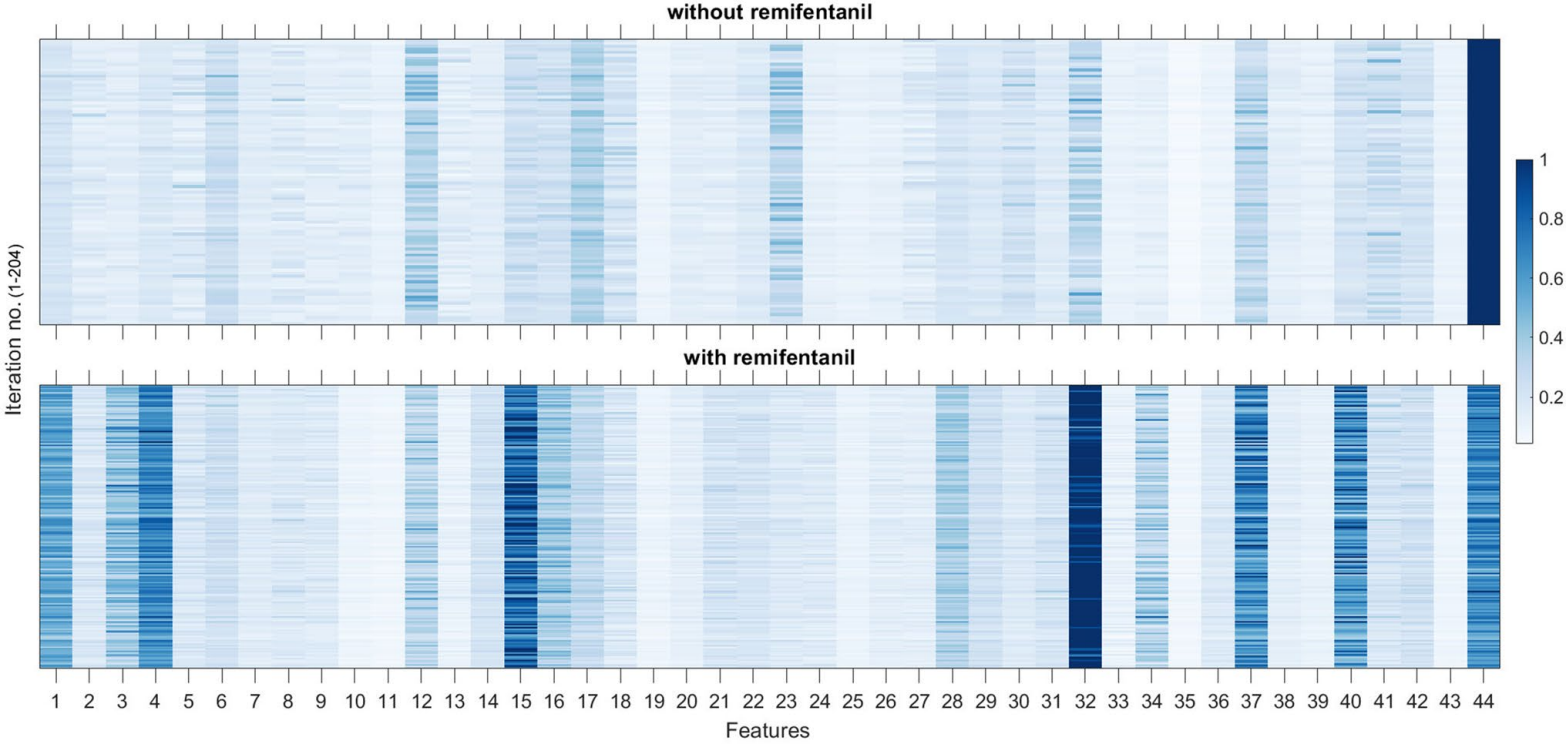
Heatmap illustrating the weights (normalized to 1) assigned by the ensemble tree with bagging algorithm. Different features were selected when remifentanil was added to propofol, sevoflurane, dexmedetomidine. Here dark blue indicates highest weight assigned by the elastic-net regularization algorithm. Fractal dimension had highest weight in both cases

Fig. 4 illustrates the heatmap of weights assigned by the ET-B algorithm to individual features across all iterations. Different features were selected in different iterations and 6 features were highly discriminatory (normalized weight ≥ 0.3) without remifentanil: BSR, *P*_*β*_, *P*_*β*_/*P*_*T*_, standard deviation of FM, SVDE, and FD. After the inclusion of remifentanil 12 features had weights above 0.3: NE, mobility, complexity, BSR, *P*_*α*_, *P*_*σ*_, *P*_*α*_/*P*_*θ*_,standard deviation of FM, kurtosis of FM, SVDE, SE, and FD. This suggests that with the addition of remifentanil, the properties of EEG change and the ET-B algorithm requires more features to achieve comparable prediction performance.

### Effect of age

To evaluate the effect of age on the performance of the ET-B model, we divided the dataset into three sub groups: group1–18 to 35 years, group 2–35 to 50 years and group 3–50 to 70 years. We then performed three different training testing combinations: (i) train on group 1 test on groups 2 and 3, (ii) train on group 2 test on groups 1 and 3 and (iii) train on group 3 test on groups 1 and 2. Table 2 summarizes the performance. We can see that the performance of the model was nearly similar when trained and tested within the same age group, however, it dropped significantly (approximately 10% reduction in the overall AUC) during cross training and testing (trained and tested on different groups).

**Table 2:** Summary of AUC’s (mean AUC (95% CI)) obtained for each model when trained and tested across different age groups. The performance significantly dropped when trained and tested across different groups demonstrating age specific nature of the sedation level prediction models. Group1 = 18–35 years; Group 2 = 35–50 years and Group 3 = 50–70 years

	Group1	Group2	Group3
Group1	0.89 (0.79–0.95)	0.75 (0.74–0.76)	0.73 (0.71–0.74)
Group2	0.77 (0.75–0.79)	0.88 (0.77–0.95)	0.80 (0.78–0.82)
Group3	0.78 (0.77–0.79)	0.83 (0.81–0.84)	0.89 (0.76–0.95)

**Table 3:** Summary of the grid search range used to tune machine learning hyperparameters. The optimal value refers to the value obtained during the training process

Model	Hyperparameter	Grid search range (min,max,step size)	Optimal parameter
EN-LR	α (Regularization)	0,1,0.1	0.9
SVM-G	γ = gaussian kernel, C = cost function	0.1, 100,0.1	γ =2.5, C = 50
RF	number of trees	50, 1000,10	500
ET-B	Number of learning cycles	10,200,5	30

Abbreviations: *EN-LR* = elastic net logistic regression; *SVM-G* = support vector machine with Gaussian kernel; *RF* =, random forest; *ET-B* = Ensemble tree with bagging

### Effect of sex

To evaluate the influence of sex, we performed cross training and testing i.e., we trained the ET-B model on male and tested it on female and vice-versa. When trained and tested within the same sex the prediction performance of the ensemble model was similar: AUC = 0.88 (0.82–0.92) and 0.90 (0.85–0.94) for male and female, respectively. However, the overall performance dropped by 9% (0.79 (0.75–0.85)) and 8% (0.82 (0.77–0.88)) for male and female, respectively during cross training and testing.

## Discussion

In recent years, there is a growing interest in developing EEG-based level of sedation monitors. However, among several unresolved important questions, it was not clear why these monitors failed to perform across different anesthetic drugs and patient groups. In this study, we compared the performance of four machine learning models trained on a large dataset of 204 EEG recordings. Using a large set of 44 QEEG features, the ensemble tree with bagging (ET-B) machine learning model achieved the best prediction performance of AUC > 0.85 to discriminate between awake and sedated states. There are four major contributions of this study: (i) we developed a technique for a drug-independent nonlinear machine learning based sedation level prediction system, (ii) we showed that individual features and/ or features derived from spectral domain are not sufficient for real-time sedation level prediction at population level, (iii) we demonstrated how addition of remifentanil affects the prediction performance of different features, and (iv) we demonstrated the importance of the inclusion of all age groups and sex to develop a robust patient-independent sedation level monitoring system.

The EEG is the only technique available to accurately monitor sedation levels in real-time. One of the main issues in developing EEG based sedation level monitors is the “feature engineering”: which features should be used to accurately predict sedation states? Current EEG based sedation level monitors either use a single feature or few expert defined spectral features to predict sedation levels [6, 8]. Additionally, the inclusion of remifentanil significantly decreased the predictive ability of all features as shown in Fig. 3. Our results suggest that neither of these approaches is ideal and a multidimensional approach together with nonlinear machine learning algorithms would be an alternate choice for developing a robust monitor.

It should be noted that we only performed binary classification to discriminate between two extreme levels of sedation: awake and sedated. If the model is not robust in this scenario, it will not be efficient to discriminate multiple levels of sedation. However, we have already developed a method to estimate continuous level of sedation from binary classification via sigmoid transformation in our previous work [14]. Except for tree based methods, we found that the performance of all other machine learning models was significantly influenced by the addition of remifentanil. ET-B is an ensemble algorithm that develops a predictive model by combining multiple decisions to decrease bias/variance via bagging or bootstrap aggregation [35]. A highly robust predictive decision is obtained by majority voting of decisions from individual classifiers in each ensemble. It was observed that the ET-B algorithm selected a different combination of features to differentiate between awake and sedated states. Only four features: BSR, standard deviation of FM, SVDE and FD were commonly selected in all conditions making it an important feature to predict sedation levels. It should be noted that only two features from the spectral domain (power in alpha band and power in beta band) were selected by the ET-B algorithm suggesting that features derived from the traditional spectral analysis alone are not sufficient to track sedation levels.

## Limitations

There are several limitations in this study. First, despite using advanced nonlinear machine learning algorithms, we did not achieve perfect discrimination between awake and sedated states (AUC = 1.0). Inclusion of additional data and/or QEEG features could help improve the performance. Second, we only used four anesthetic drugs in this study. Validation on another external dataset with combination of multiple drugs is required to explore the robustness of the proposed system. Third, we did not include pediatric (< 18 years) and data from elderly cohorts (>70 years) in this analysis due to the nature of the clinical trial. Fourth, we only used data from healthy volunteers which may not reflect the influence of disease severity/routine medications on the EEG.

## Conclusion

Despite the above mentioned limitations, the findings in this study suggests that by pooling data from different drugs, age and sex groups, it is possible to develop a robust realtime sedation level prediction system using advanced nonlinear machine learning algorithms. Features derived from traditional spectrogram alone are not sufficient to accurately predict levels of sedation. It is hoped that findings in this study would help understand the mechanism of anesthetics/sedatives on EEG and help in developing improved and robust sedation level monitoring systems.

## Data Availability

Due to the nature of the clinical trial and regulations, the dataset described in this manuscript cannot be made available.

## Code availability

The MATLAB code used in this study can be made available to interested researchers on a reasonable request to the corresponding author.
